# Combined Malonic and Methylmalonic Aciduria Due to *ACSF3* Variants Results in Benign Clinical Course in Three Chinese Patients

**DOI:** 10.3389/fped.2021.751895

**Published:** 2021-11-25

**Authors:** Ping Wang, Jianbo Shu, Chunyu Gu, Xiaoli Yu, Jie Zheng, Chunhua Zhang, Chunquan Cai

**Affiliations:** ^1^Tianjin Pediatric Research Institute, Tianjin Children's Hospital (Tianjin University Children's Hospital), Tianjin, China; ^2^Tianjin Key Laboratory of Birth Defects for Prevention and Treatment, Tianjin, China; ^3^Graduate College of Tianjin Medical University, Tianjin, China; ^4^Department of Neurology, Tianjin Children's Hospital (Tianjin University Children's Hospital), Tianjin, China; ^5^Matsumoto Institute of Life Science (MILS) International, Yokohama, Japan; ^6^Department of Neurosurgery, Tianjin Children's Hospital (Tianjin University Children's Hospital), Tianjin, China

**Keywords:** combined malonic and methylmalonic aciduria, *ACSF3* gene, benign condition, Chinese population, novel variant

## Abstract

**Introduction:** Combined malonic and methylmalonic aciduria (CMAMMA) is a rare metabolic disease caused by biallelic variants in *ACSF3* gene. The clinical phenotype is highly heterogeneous in this disorder, ranging from asymptomatic to severe symptoms. No cases with CMAMMA were reported in China.

**Materials and Methods:** In this study, three Chinese pediatric patients were diagnosed with CMAMMA unexpectedly while being treated for other ailments. To better characterize CMAMMA in a Chinese population, we made a multidimensional analysis with detailed clinical phenotype, semi-quantitative detection of urine organic acid, and analysis of *ACSF3* gene variants.

**Results:** The clinical presentation of these patients is quite different; their main complaints were anemia, jaundice, or abnormal urine test, respectively. They showed no symptoms of the classic methylmalonic academia, but urine organic acid analysis showed elevated malonic acid and methylmalonic acid in all the patients repeatedly. Variants were found at four sites in *ACSF3* gene. Patient 1 carried the compound heterogeneous variant c.689G> A (p.Trp230^*^)/c.1456G> A (p.Ala486Thr). A compound heterozygous variant c.473C> T (p.Pro158Leu)/c.1456G> A (p.Ala486Thr) was identified in patient 2. Patient 3 harbored a novel homozygous variant c.1447A> G (p.Lys483Glu).

**Conclusions:** Three Chinese patients were diagnosed with CMAMMA caused by *ACSF3* variants. Their clinical course revealed that CMAMMA can be a benign condition that does not affect individual growth and development, but severe clinical phenotype may appear when other triggers exist. This study systematically elaborates CMAMMA in a Chinese population for the first time, broadens the spectrum of gene variant, and provides a strong basis for the etiological study of this disorder.

## Introduction

Methylmalonic academia (MMAemia) is a common inborn error of metabolism characterized by abnormal accumulation of methylmalonic acid in body fluids, resulting in many serious clinical manifestations. MMAemia is mainly caused by the defect of methylmalonyl-CoA mutase (MUT) or its coenzyme cobalamin (Cbl) (1). Combined malonic and methylmalonic aciduria (CMAMMA) is a rare atypical form of MMAemia featured with increased concentrations of malonic acid (MA) and methylmalonic acid (MMA) in urine (2). CMAMMA is caused by biallelic variants in the AcylCoA synthetase family member 3 (*ACSF3*, OMIM:614265) gene. *ACSF3* encodes a mitochondrial acyl-CoA synthetase, which is essential for the synthesis of malonyl-CoA as well as methylmalonyl-CoA (3). Patients who carried *ACSF3* gene variants excrete more MMA than MA in reported cases, which make them distinct from the patients with classical MMAemia (4).

The gene of *ACSF3* is located on chromosome 16q24.3. It consists of 11 exons and encodes a 576-amino-acid protein with the first 83 residues representing the predicted mitochondrial transit peptide (4). To date, more than 50 patients worldwide have been reported to have CMAMMA caused by homozygous or compound heterozygous variants in *ACSF3*, including missense, nonsense, deletion, frameshift, and splice site variants. The clinical presentation of CMAMMA is quite controversial. Signs and symptoms reported so far are involved in neurological abnormalities in adults and infection induced encephalopathy in pediatric patients. Nevertheless, asymptomatic patients with normal outcomes strongly suggest that CMAMMA can be a benign state (2, 4–9). No case of the disease has been reported in China so far.

In this study, we summarized the clinical course, urine organic acid screening results, and analysis of *ACSF3* gene variants in three Chinese patients with CMAMMA diagnosed in our hospital. We identified four variants inherited from the parents separately, among which c.1447A> G (p.K483E) is a novel variant. Based on the present study and literature review, we speculate that the CMAMMA is a benign state when other triggers are absent in Chinese population.

## Materials and Methods

### Participants

Three patients at the age of 11 days to 8 months were diagnosed as CMAMMA in Tianjin Children's Hospital in China. There was no family history in these patients. The informed consents from guardians and the approval of the Medical Ethics Committee of Tianjin Children's Hospital were obtained.

### Routine Tests and Metabolic Assay

Routine tests were conducted in three patients, such as physical examination, routine blood, urine and stool test, blood gas analysis, biochemical test, imaging examination, and etiological examination for infected patients. Semi-quantitative analysis of organic acid in urine was performed using gas chromatography–mass spectrometry (GC/MS).

### Whole Exome Sequencing and Analysis

The peripheral blood samples were collected to extract the genomic DNA. Peripheral blood from the patients and their parents were collected. A Blood Genomic DNA Mini kit was used to extract the genomic DNA according to the manufacturer's protocol. WES was performed to detect the pathogenic gene variants. Paired-end sequencing was performed on more than 95% of the target regions, with a read length of 150 bp and an average coverage depth of 100-fold, covering all coding regions and exon–intron boundaries. The Burrows-Wheeler Aligner (BWA) software was used to align the raw data with the human reference genome hg19. Insertions, deletions, and single-nucleotide polymorphism sites (SNPs) were analyzed by the Genome Analysis Tool Kit (GATK) software. Annovar software was utilized to add the annotated information of databases such as HGMD, DBSNP, OMIM, and 1000 Genomes. Protein function was predicted by SIFT and Polyphen2 software. The filtering of the variants was performed based on the phenotype, gene frequency, variant type, inherited pattern, and bioinformatics analysis. Suspected variants were validated by Sanger sequencing.

## Results

### Clinical Data

Patient 1 was a 3-month-old girl. She was admitted to the hospital due to pale face for 2 weeks, fever, and cough for 2 days. Detailed physical examination and laboratory tests revealed that the patient had anemia, splenomegaly, hepatomegaly, thrombocytopenia, and bronchitis. Widened interval outside the cerebrum was noted in computed tomography. These clinical features were mainly attributed to suspected Evans syndrome and viral infection. The patient is 5 years old now and shows no sign of physical or psychomotor retardation.

Patient 2 was an 11-day-old boy. He was born at term by normal delivery. He was referred to the hospital due to jaundice for 7 days. Detailed physical examination showed neonatal omphalitis, myocardial damage, testicular hydrocele, and liver function damage. Laboratory tests showed that the direct bilirubin level was 17.1 μmol/L (normal: 0–5 μmol/L), and indirect bilirubin level was 184.4 μmol/L (normal: 3.4–10.3 μmol/L). These clinical manifestations were mainly caused by jaundice and infection. He is 3 years old now and attains a development milestone.

Patient 3 was an 8-month-old boy. He was born at term by cesarean section. He was admitted because of abnormal urine test for 3 months with red urine and urinary frequency. Urinary tract infection, pneumonia, and diarrhea were found in this patient. No obvious abnormality in his growth development was present. Infection factors were the main cause of his clinical presentations. He shows no sign of psychomotor retardation at the age of 5 in our recent follow-up.

### Urine Analysis

**Figure 1:**
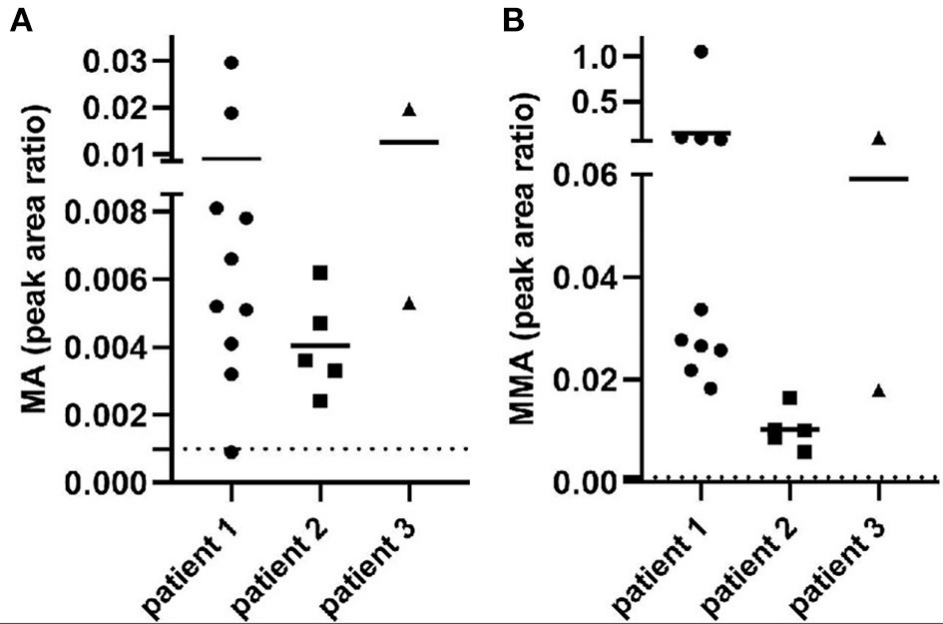
MA and MMA excretion in urine from all the patients. The *x*-axis represents different patients, and the value of the *y*-axis means metabolite peak area ratio to creatinine (Cr). Dotted lines represent reference range. **(A)** Peak area ratio of MA/Cr indicated the MA excretion in patients. **(B)** Peak area ratio of MMA/Cr indicated the MMA excretion in patients.

GC/MS demonstrated elevated MA and MMA in all the patients repeatedly (Figure 1), which suggested the suspected diagnosis of MMAemia. However, the patients did not present with the clinical symptoms of classic MMAemia.

### Gene Variation Analysis

**Figure 2:**
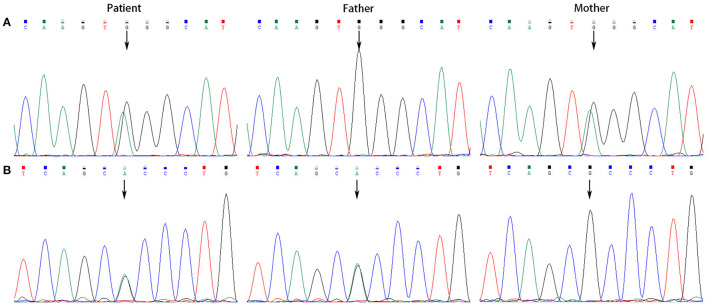
Sanger sequencing of the genomic DNA from patient 1 and her parents. **(A)** Patient 1 carried with a heterozygous variant of c.689G> A, her father with a normal genotype, and mother with a heterozygous variant of c.689G> A. **(B)** Patient 1 carried with a heterozygous variant of c.1456G> A, her father with a heterozygous variant of c.1456G> A, and mother with a normal genotype.

**Figure 3:**
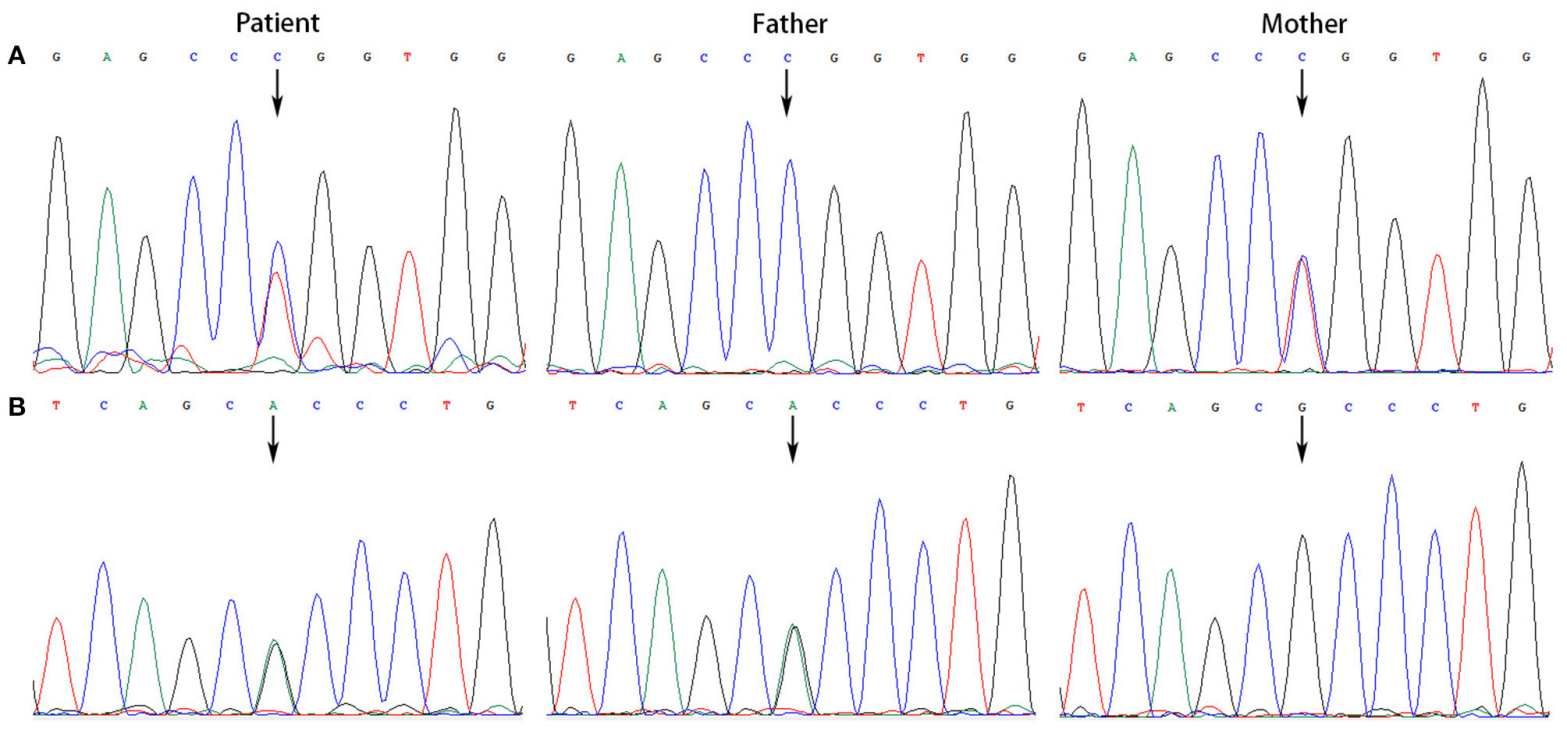
Sanger sequencing of the genomic DNA from patient 2 and his parents. **(A)** Patient 2 with a heterozygous variant of c.473C> T, his father with a normal genotype, and mother with a heterozygous variant of c.473C> T. **(B)** Patient 2 with a heterozygous variant of c.1456G> A, his father with a heterozygous variant of c.1456G> A, and mother with a normal genotype.

**Figure 4:**
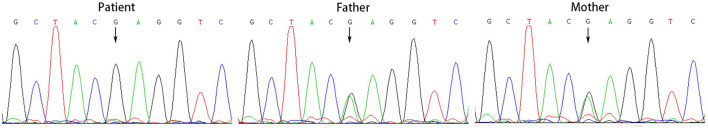
Sanger sequencing of the genomic DNA from patient 3 and his parents. Patient 3 harbored a homozygous variant of c.1447A> G in *ACSF3* gene, both his father and mother carried this variant.

All the patients were detected to carry variants in *ACSF3* gene (NM_174917.4) by WES. Patient 1 carried a compound heterozygous variant. The variant c.689G> A (p.Trp230^*^) was inherited from her mother and the other variant c.1456G> A (p.Ala486Thr) was inherited from her father. Besides, the compound heterozygous variant, c.473C> T (p.Pro158Leu)/c.1456G> A (p.Ala486Thr), was identified in the *ACSF3* gene in patient 2. The two heterozygous variants were inherited from the mother and father, respectively. Patient 3 harbored a homozygous variant of c.1447A> G (p.Lys483Glu) in *ACSF3* gene, which was inherited from his parents (Table 1). Sanger sequencing was used to confirm the variants (Figures 2–4). The homozygous variant c.1447A> G in *ACSF3* gene is a novel discovery.

**Table 1:** Genetic and phenotypic finding of patients with CMAMMA.

**Patient**	**Age and sex**	**Clinical feature**	**Variants**		**References**
1	3mo, F	Anemia, splenomegaly, hepatomegaly, thrombocytopenia, and bronchitis	c.689G> A^a^ (p.W230*)	c.1456G> A^b^ (p.A486T)	Present study
2	11d, M	Jaundice, neonatal omphalitis, myocardial damage, testicular hydrocele, and liver function damage	c.473C> T^a^ (p.P158L)	c.1456G> A^b^ (p.A486T)	Present study
3	8mo, M	Urinary tract infection, pneumonia, and diarrhea	c.1447A> G^a^ (p.K483E)	c.1447A> G^b^ (p.K483E)	Present study
4	14y, M	Asymptomatic	c.1411C> T (p.R471W)	c.1411C> T (p.R471W)	(4)
5	4y, F	Asymptomatic	c.1075G> A (p.E359K)	c.1075G> A (p.E359K)	(4)
6	2y, M	Asymptomatic	c.1075G> A (p.E359K)	c.1075G> A (p.E359K)	(4)
7	43y, F	Ocular migraine, memoryproblems, T2 hyperintensities on brain MRI	c.1385A> C (p.K462T) c.del1394_1411 (p.Q465_G470)	c.1672C> T (p.R558W)	(2)
8	51y, M	Complex partial seizures, and memory problems	c.1567C> T (p.R523*)	c.1672C> T (p.R558W)	(2)
9	55y, F	Psychiatric symptoms, T2 hyperintensities on brain MRI	c.1075G> A (p.E359K)	c.1672C> T (p.R558W)	(2)
10	22mo, F	Seizure, encephalopathy, ketoacidosis	c.1672C> T (p.R558W)	c.1672C> T (p.R558W)	(2)
11	4y, F	Hypoglycemia, acidosis, poor weight gain, diarrhea episodes	c.1073C> T (p.T358I)	c.1412G> A (p.R471Q)	(2)
12	66y, F	Incontinence, mild memory problems	c.1411C> T (p.R471W)	c.1411C> T (p.R471W)	(2)
13	6mo, M	Failure to thrive, elevated transaminases	c.728C> T (p.P243L)	c.728C> T (p.P243L)	(2)
14	17mo, M	Psychomotor delay, microcephly, dystonia, axial hypotonia, and speech delay	c.593T> G (p.M198R)	c.593T> G (p.M198R)	(2)
15	26mo, M	Psychomotor delay, hypotonia, and loss of speech	–	–	(2)
16	6y, F	Asymptomatic until 6y, encephalopathic event upon an influenza infection	c.1075G> A (p.E359K)	c.311A> T (p.N104I)	(5)
17	5.5y, F	Asymptomatic until 5.5y, encephalopathic event upon an influenza infection	c.1075G> A (p.E359K)	c.311A> T (p.N104I)	(5)
18	2y, F	Mild developmental delay and seizure disorder	c.1672C> T (p.R558W)	c.1673G> A (p.R558Q)	(6)
19	73y,M	Late-onset neurologic syndrome, rare axonal degeneration of segmental myelin thinning.	c.634G> C (p.V212L)	c.781G> T (p.G261*)	c.854C> T (p.P285L)	(6)
20	10y, F	Significant developmental and speech delays	c.1470G> C (p.E490D)	c.1470G> C (p.E490D)	(6)
21	8mo, F	Persistent elevation of MMA	c.1239+2T> G	c.1672C> T (p.R558W)	(6)
22	3y, F	Recurrentvomiting, febrile seizures	Chr16:87441993:89171912deletion (spanning *ACSF3*)		c.1672C> T (p.R558W)	(6)
23	n/a	Asymptomatic at 3y	c.1446_1447delCA (p.Y482*)	c.424C> T (p.Q142*)	(7)
24	n/a	Hearing loss, psychomotor delay	c.1075G> A (p.E359K)	c.1075G> A (p.E359K)	(7)
25	n/a	Seizures, dystonia	c.1075G> A (p.E359K)	c.1672C> T (p.R558W)	(7)
26	n/a	Asymptomatic at 7 m	c.820C> T (p.Q274*)	c.820C> T (p.Q274*)	(7)
27	2y, M	Hydronephrosis, several renal cysts	c.1718delT	c.1672C> T (p.R558W)	(8)
28	7y, F	None	n/a	n/a	(8)
29	6y, F	None	c.1446_1447delCA (p.Y482*)	c.1075G> A (p.E359K)	(8)
30	24y, F	None	c.1075G> A (p.E359K)	c.1470G> C (p.E490D)	(8)
31	5y, F	Growth at 3rd percentile, consistent with parental heights, allergic rhinitis, eczema	c.1239+2T> G	c.1672C> T (p.R558W)	(8)
32	2y, M	Growth at 3–15th percentile, consistent with parental heights	n/a	n/a	(8)
33	11y, M	Hypopigmented spots	n/a	n/a	(8)
34	27y, F	Recurrent urinarytract infection, hives, and endometriosis	c.1672C> T (p.R558W)	c.1672C> T (p.R558W)	(8)
35	24y, M	Hepatomegaly and bovine protein intolerance in infancy, bilateral vesicoureteral reflux	c.1075G> A (p.E359K)	c.1075G> A (p.E359K)	(8)
36	22y, F	Attention deficit hyperactivity disorder	n/a	n/a	(8)
37	20y, M	Nissen fundoplication for GERD in infancy	n/a	n/a	(8)
38	23y, F	Neonatal cutaneous lupus erythematosus	c.1553C> A (p.A518D)	c.1553C> A (p.A518D) c.473C> T (p.P158L)	(8)
39	20y, F	Slightly smaller right kidney, and migraines	n/a		n/a	(8)
40	7y, M	None	c.1075G> A (p.E359K)	c.1672C> T (p.R558W)	(8)
41	13y, M	None	c.1075G> A (p.E359K)	c.1672C> T (p.R558W)	(8)
42	6 mo, F	Severe bronchiolitis/pneumonia at 1 month, lactic acidosis, mild valvular pulmonary stenosis, height and weight 0.1–3^rd^ percentile	c.1075G> A (p.E359K)	c.1075G> A (p.E359K)	(8)
43	7y, M	None	c.774_775del (p.W259Gfs*10)	c.1672C> T (p.R558W)	(8)
44	14y, M	Attention deficit hyperactivity disorder, dysorthography	c.1470G> C (p.E490D)	c.1081G> A (p.G361S)	(8)
45	16y, M	Eczema, scoliosis, glaucoma and cataract, mild language and fine motor delay in 1st years, corrected by school age, cyclic vomiting in adolescence with no metabolic derangement	c.1239+2T> G	c.1075G> A (p.E359K)	(8)
46	30y, F	Dehydration and metabolic acidosis in the context of severe bloody diarrhea at 4 weeks of age, otherwise healthy	c.1075G> A (p.E359K)	c.1075G> A (p.E359K)	(8)
47	19y, M	Attention deficit hyperactivity disorder	c.1411C> T (p.R471W)	c.1411C> T (p.R471W)	(8)
48	8y, F	Bilateral congenital dacryostenosisy	c.1075G> A (p.E359K)	c.1075G> A (p.E359K)	(8)
49	7y, M	None	c.1075G> A (p.E359K)	c.1075G> A (p.E359K)	(8)
50	6y, M	None	c.1075G> A (p.E359K)	c.1672C> T (p.R558W)	(8)
51	7y, M	Latent nystagmus	c.634G> A (p.V212 M)	c.1470G> C (p.E490D)	(8)
52	6y, M	Neonatal jitteriness, developmental delay, Autism, Joint hypermobility	c.1453A> C (p.S485R)	c.1453A> C (p.S485R)	(9)

*mo, month; d, day; y, year*;

a *variant was inherited from mother*;

b *variant was inherited from father*;

*–, no variant in coding exons or splice sites; n/a, not available*.

## Discussion

**Figure 5:**
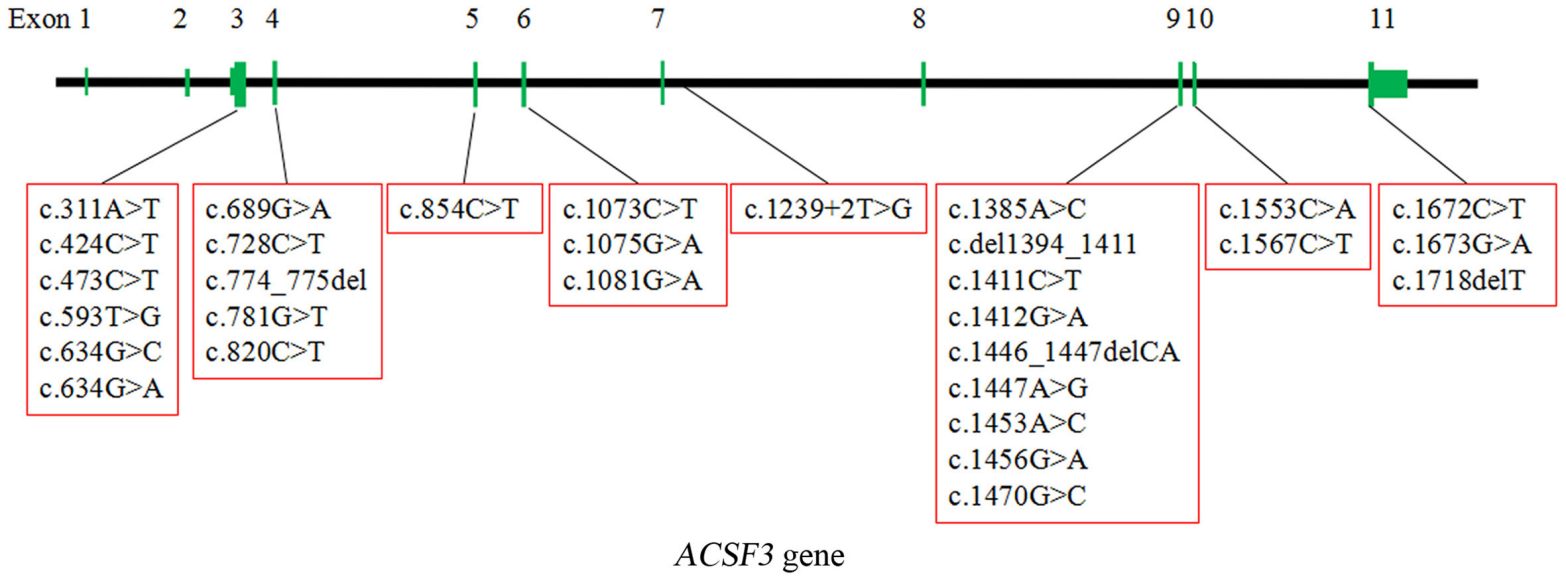
The localization of 30 homozygous or compound heterozygous variants in *ACSF3* reported worldwide (except for a Chr16:87441993:89171912 deletion spanning *ACSF3*). The types of the variants include missense, nonsense, deletion, frameshift, and splice site variants. The most common type is missense.

CMAMMA is a rare inborn error of metabolism characterized by high excretion of MMA than MA in urine. The pathogenic gene *ACSF3* catalyzes the initial reaction in intramitochondrial fatty acid synthesis by activating MA and MMA into their respective CoA thioesters. To date, about 52 patients with 31 homozygous or compound heterozygous variants of *ACSF3* have been reported worldwide, including missense, nonsense, deletion, frameshift, and splice site variants. The locations of these variants in *ACSF3* are graphically displayed in Figure 5. Most of the variants are missense, mainly located at the carboxyl terminal of the protein (Table 1) (2, 4–9). The most common *ACSF3* variants reported so far are c.1075G> A (p.E359K) and c.1672C> T (p.R558W), which are also the most common variants in asymptomatic patients. It indicates that the two variants are more likely to have a mild effect on gene function. Patients who carried the heterozygous variant c.1470G> C (p.E490D) have mild clinical symptoms, while the homozygous variant can result in significant developmental and speech delays. In the present work, we identified four variants of the *ACSF3* gene (one nonsense and three missense mutations) in three Chinese patients from three unrelated families, among which the variant c.1447A> G (p.K483E) is novel. The heterozygous variant c.473C> T (p.P158L) has also been reported in another patient with benign clinical presentation (8).

Patients who carried *ACSF3* variants exhibit controversial clinical phenotypes. Based on the literature review we did, severe clinical manifestations are mainly reported in adults, with the most common symptoms of neurological problems and psychiatric features (2). Because of the long-term damage accumulation, older people are at high risk for neurological illness even without *ACSF3* variants. The phenotype of pediatric patients is relatively mild or even asymptomatic. Three pediatric patients diagnosed with CMAMMA due to *ACSF3* mutation were reported to be clinically asymptomatic, and they had age-appropriate development (4). A retrospective study described the course of 25 CMAMMA individuals and suggested that CMAMMA is probably a benign condition (8). Another study showed that infection factors could provoke metabolic dysregulation in pediatric patients with CMAMMA, and normal levels of development could be obtained at follow-up (5). Therefore, CMAMMA may be considered as a risk factor instead of a disease, which could lead to clinical symptoms when other influencing factors exist.

*ACSF3* variants that were only reported in patients with benign manifestations include c.689G> A, c.1456G> A, c.473C> T, c.1447A> G, c.311A> T, c.1239+2T> G, c.1446_1447delCA, c.424C> T, c.820C> T, c.1553C> A, c.774_775del, and c.1081G> A. Patients carrying these variants were asymptomatic or had infection-induced symptoms that disappeared after treatment (2, 4–9). Besides, although the variant c.1456G> A has a high frequency in population, our present study showed that patients carrying this variant had repeatedly elevated biochemical phenotype without obvious clinical phenotype, which could also be observed in other inherited metabolic disease. The β-ureidopropionase deficiency patients carrying the homozygous or compound heterozygous c.977G> A variant (high prevalence in normal Japanese population) in *UPB1* gene could be asymptomatic as well (10). We speculate that the c.1456G> A variant in *ACSF3* may act in a similar way. High frequency of c.1456G> A in population indicates that CMAMMA may not be as rare as generally considered, and this kind of variant may contribute to the benign condition in patients. Accumulations of free MA and MMA were neurotoxic *in vitro*, while the concentrations might be insufficient to produce disease in CMAMMA patients (8). Actually, we cannot completely exclude the possibility that the accumulation of metabolites is associated with the occurrence of neurological symptoms as the patients grow up, or that the people with the *ACSF3* gene variants are more genetic susceptible to neurological problems later in life. Increased dependency on β-oxidation for energy production were observed in fibroblasts from CMAMMA patients. As a result, the subsequent increased risk for hypoxia and oxidative stress may be crucial for the onset of neurological symptoms in the long run (11).

## Conclusions

In conclusion, our study included three Chinese pediatric patients under the age of 4 years. Their clinical features were mainly caused by infection or other reasons. They showed no sign of physical or psychomotor retardation in our follow-up. Combined with reported cases, our study strongly suggested that the CMAMMA is a benign clinical course, especially for patients carrying certain variants. In addition, the present study had some limitations. Firstly, this study only involved the correlation analysis between genotype and phenotype. Functional verification of variants *in vitro* will help to elucidate the pathogenesis of CMAMMA, especially for the novel variant c.1447A> G (p.K483E), while functional studies were not available in this study due to the limitations of experimental condition. Secondly, a longer follow-up period is needed to better assess the growth and development of the patients.

## Data Availability Statement

The original contributions presented in the study are included in the article/supplementary material, further inquiries can be directed to the corresponding author/s.

## Ethics Statement

The studies involving human participants were reviewed and approved by the Medical Ethics Committee of Tianjin Children's Hospital. Written informed consent to participate in this study was provided by the participants' legal guardian/next of kin.

## Author Contributions

PW and JS conceived the concept and wrote the manuscript. CG contributed to literature review and revised the manuscript. XY provided clinical diagnosis. JZ contributed to interpretation of the results. CZ performed the analysis. CC participated in supervision of the project. All authors read and approved the final manuscript.

## Funding

This research was supported by the National Natural Science Foundation of China (81771589), the Program of Tianjin Science and Technology Plan (18ZXDBSY00170), and the Public Health and Technology project of Tianjin (KJ20166, ZC20120).

## Conflict of Interest

The authors declare that the research was conducted in the absence of any commercial or financial relationships that could be construed as a potential conflict of interest.

## Publisher's Note

All claims expressed in this article are solely those of the authors and do not necessarily represent those of their affiliated organizations, or those of the publisher, the editors and the reviewers. Any product that may be evaluated in this article, or claim that may be made by its manufacturer, is not guaranteed or endorsed by the publisher.
